# The course of chemotherapy-induced peripheral neuropathy (CIPN) in hematological patients treated with vincristine, bortezomib, or lenalidomide: the NOVIT study

**DOI:** 10.1007/s00520-025-09282-3

**Published:** 2025-02-26

**Authors:** Eva Futtrup Maksten, Carsten Dahl Mørch, Lasse Hjort Jakobsen, Kristian Hay Kragholm, Pernille From Blindum, Mikkel Runason Simonsen, Niels Ejskjaer, Karen Dybkær, Henrik Gregersen, Jakob Madsen, Tarec C. El-Galaly, Marianne Tang Severinsen

**Affiliations:** 1 Research Section, Department of Hematology, Clinical Cancer Research Center, Aalborg University Hospital, Sdr. Skovvej 15, 9000 Aalborg, Denmark; 2 Department of Clinical Medicine, Aalborg University, Aalborg, Denmark; 3 Center for Neuroplasticity and Pain (CNAP), Center for Sensory-Motor Interaction, Department of Health Science and Technology, Aalborg University, Aalborg, Denmark; 4 Department Mathematical Sciences, Aalborg University, Aalborg, Denmark; 5 Department of Cardiology, Aalborg University Hospital, Aalborg, Denmark; 6 Steno Diabetes Center North Denmark and Department of Endocrinology, Aalborg University Hospital, Aalborg, Denmark; 7 Department of Hematology, Odense University Hospital, Odense, Denmark; 8 Clinical Epidemiology Division, Department of Medicine, Karolinska Institute, Stockholm, Sweden

**Keywords:** Tingling, Numbness, Survivorship, Lymphoma, Multiple myeloma

## Abstract

**Purpose:**

To assess and describe chemotherapy-induced peripheral neuropathy (CIPN), a well-known complication to cancer treatment, using different methodologies in hematological patients.

**Methods:**

Patients scheduled for treatment with vincristine, bortezomib, or lenalidomide were included in this longitudinal observational study. The patients were examined for CIPN before treatment (baseline), before each chemotherapy cycle, one month after end of treatment, and one year after baseline using patient-reported outcomes (Functional Assessment of Cancer Therapy/Gynecologic Oncology Group-Ntx-13 (FACT/GOG-Ntx-13)) and clinician-assessed outcomes (the Common Terminology Criteria for Adverse Events (CTCAE) and the Total Neuropathy Score-clinical version (TNSc©)).

**Results:**

A total of 23 patients with 171 examination visits were included between 2020 and 2022. Four patients were treated with vincristine, five with bortezomib, and fourteen with bortezomib and lenalidomide combined. Defining CIPN as a ≥ 10% decrease in the FACT/GOG-Ntx-13, 11 patients (47.8%) developed CIPN during treatment and follow-up. CTCAE score for paresthesia increased from baseline throughout treatment until 1 month after the last treatment (p ≤ 0.045). Overall, the highest proportion of CIPN was present at cycle 3–4 and 1 month after last treatment.

**Conclusion:**

This study describes the course of CIPN in patients treated with vincristine, bortezomib, or lenalidomide using both patient-reported and clinician-assessed outcomes. The highest proportion of CIPN was present at cycle 3–4 and 1 month after treatment, at which timepoints clinicians must be especially aware of CIPN.

**Trial registration:**

Registered at Clinicaltrials.gov (Trial Registration Number: NCT04393363) on March 19, 2020.

**Supplementary Information:**

The online version contains supplementary material available at 10.1007/s00520-025-09282-3.

## Introduction

Chemotherapy-induced peripheral neuropathy (CIPN) is a common but not fully understood side effect to cancer treatment. It can cause acute and chronic damage to both sensory, motoric, and autonomic nerve fibers. The neuropathy is length-dependent with hands and feet being the main site of symptoms [1–4]. CIPN affects 30–40% of all patients after treatment with chemotherapy; however, incidence up to 85% has been reported [3, 5, 6]. The key issue of measuring the incidence and prevalence of CIPN is the absence of a gold standard for the assessment of CIPN. It is furthermore complicated by differences in risk of CIPN in different types of chemotherapies, and symptoms of neuropathy due to other reasons, e.g., diabetes mellitus. Besides, CIPN symptoms can be reversible, increase or decrease during treatment or even develop after treatment (the coasting phenomenon) which makes it difficult to choose the right time for assessment [7]. Finally, some patients may be reluctant to pronounce symptoms evolved during treatment, because they fear changes in treatment that could affect their survival [8]. This could lead to symptoms that could have been avoided if detected earlier. Symptoms of CIPN can have a large impact on everyday life and result in functional disability and emotional distress [9, 10]. Management of CIPN is difficult. Normally, symptoms during treatment with chemotherapy are handled with dose reduction. Various pharmacological and non-pharmacological prevention and treatment modalities have been tested with duloxetine as the treatment being the most extensively examined. Other treatment options included exercise therapy, acupuncture, scrambler therapy, gabapentin/pregabalin, tricyclic antidepressant, and venlafaxine. However, the utility of the latter options all requires further validation. The prevention possibilities include cryotherapy, compression-, and exercise therapy. Again, further validation is needed to confirm their efficacy [11, 12].

As early detection is essential and the burden of CIPN may fluctuate some studies have assessed the prevalence and course of CIPN during treatment. Wang et al. and Molassiotis et al. examined patients with breast cancer and mixed cancer types treated with taxanes- and platinum-based chemotherapy. Examinations were performed before each new treatment cycle and after treatment, and they found the highest prevalence of CIPN at the end of treatment [13, 14]. Studies examining the course of CIPN in patients treated with bortezomib have a low frequency of examinations and only report clinician-assessed CIPN or an overall result at the end of treatment thereby missing the opportunity to describe the course of CIPN in detail. Others focus on information from medical charts and diagnosis of neuropathy, which may underestimate the risk of CIPN [15–20].

To our knowledge no prior study has prospectively studied the course of CIPN before, at each chemotherapy cycle, and after treatment in hematological patients treated with either vincristine, bortezomib, or lenalidomide using different methodology including both patient- and clinician-assessed methods. The aim of this study was to assess the prevalence of CIPN and to evaluate several subjective and objective methods for quantification and grading of CIPN in patients with a hematological malignancy scheduled for treatment with either vincristine, bortezomib, or lenalidomide. All measurements were performed before, during, and after treatment, to identify the time of highest CIPN risk, and to find the most suitable instrument for detection of CIPN in a clinical setting.

## Methods

### Study population

Patients with lymphoma or multiple myeloma, who were scheduled for treatment with vincristine, bortezomib, or lenalidomide were invited to participate in this observational study conducted between August 31, 2020, and August 1, 2023, at Aalborg University Hospital, Denmark.

Patients fulfilling the following in- and exclusion criteria were included: age ≥ 18 years; not started treatment with vincristine, bortezomib, or lenalidomide before inclusion (< 2 weeks treatment with lenalidomide was accepted); signed informed consent form; not diagnosed with vitamin B12 deficiency and treated within the last year; not diagnosed with neural damage, severe diseases in the neural system, or severe skin disease; and not having a pacemaker or other implanted electronic medical devices. Even though diabetes increases the risk of neuropathy, patients were allowed to have preexisting diabetes to reflect a real-world setting.

Treatment with vincristine, bortezomib, or lenalidomide was given in four different regimens: (a) R-CHOP (rituximab, cyclophosphamide, doxorubicin, vincristine, and prednisolone) with 2 mg vincristine given every 21th day in 6 cycles, (b) VRd (bortezomib, lenalidomide, and dexamethasone) in a modified regimen for elderly patients including 6–8 cycles given 28 days apart with bortezomib (1.3 mg/m^2^) at day 1, 8, and 15 and 25 mg lenalidomide every or every second day for 21 days, (c) VRd and HDT-ASCT (bortezomib, lenalidomide, and dexamethasone and high-dose therapy-autologous stem-cell transplantation) including 4 cycles given 21 days apart with bortezomib (1.3 mg/m^2^) at day 1, 4, 8, and 11 and 25 mg lenalidomide daily for 14 days followed by HDT-ASCT approximately 1 month after end of treatment with VRd, and (d) D-VMP (daratumumab, bortezomib, melphalan, and prednisolone) including 9 cycles given 42 days apart with bortezomib (1.3 mg/m^2^) at day 1, 8, 22, and 29. Bortezomib was given subcutaneous in all regimens. Most patients with multiple myeloma were afterwards treated with maintenance treatment with 10 mg lenalidomide day 1–21 repeated every 28th day.

Patients were examined for CIPN at a visit before treatment cycle 1 (baseline), before each subsequent treatment cycle, 1 month after last treatment and finally 1 year after baseline (Table 1). The number and time of visits were adapted to the individual treatment plans and changes in scheduled treatments. The visits were performed before treatment on the day of a new treatment cycle or the weekday before. If patients were scheduled for HDT-ASCT, a visit was performed the day before the cyclophosphamide priming. The visits after end of treatment were performed 1 month after the beginning of the last chemotherapy cycle or HDT-ASCT (± 1 week) and 1 year after start of treatment (± 2 weeks).

**Table 1:** Examinations included within the study. Patient-reported outcomes include Functional Assessment of Cancer Therapy/Gynecologic Oncology Group-Ntx-13 (FACT/GOG-Ntx-13). Clinician-assessed outcomes include Total Neuropathy Score-clinical (TNSc) and Common Terminology Criteria for Adverse Events (CTCAE). Number of cycles were adjusted according to each patient’s treatment regimen and individual treatment plan

	Cycle 1 (baseline)	Cycle 2	Cycle 3	Cycle 4	Cycle 5	Cycle 6	Cycle 7	Cycle 8	Cycle 9	HDT-ASCT	1 month after last treatment	1 year after baseline
Patients treated without HDT-ASCT												
FACT/GOG-Ntx	•	•	•	•	•	•	•	•	•		•	•
CTCAE	•	•	•	•	•	•	•	•	•		•	•
TNSc	•	•	•	•	•	•	•	•	•		•	•
Patients treated with HDT-ASCT												
FACT/GOG-Ntx	•	•	•	•						•	•	•
CTCAE	•	•	•	•						•	•	•
TNSc	•	•	•	•						•	•	•

Standard blood samples at baseline, 1 month after last treatment, and 1 year after baseline were supplemented with test of cobalamin (B12), methylmalonic acid (MMA), and folate to ensure that neuropathy was not caused by vitamin deficiency.

### Patient-reported outcomes

At each visit, the patients completed the questionnaire Functional Assessment of Cancer Therapy/Gynecologic Oncology Group-Ntx-13 (FACT/GOG-Ntx-13, version 4) regarding their well-being within the last seven days [21, 22]. The questionnaire is divided into five subscales with the first four subscales (including a total of 27 questions) covering quality of life (QoL) and the last subscale (including 13 questions) covering neuropathy. The answers were graded on a five-point Likert-type scale from 0 (not at all) to 4 (very much) and converted using the scoring manual given a total score of 0–160 of which 108 points describe QoL and 52 points describe neuropathy. A decrease in score equals worsen of either QoL or neuropathy. A ≥ 10% decrease from baseline (or from the visit before cycle 2 if baseline information was missing) in the subscale regarding neuropathy (hereafter referred to as NTX-13 score) was defined as CIPN in accordance with others [23, 24]. A ≥ 10% decrease in the shortened NTX-4 (including the first four sensory questions from the NTX-13) was also examined [22].

### Clinician-assessed neuropathy

Patients were examined by two different methods to detect clinician-assessed CIPN using the Common Terminology Criteria for Adverse Events (CTCAE) and the Total Neuropathy Score-clinical version (TNSc © 2008, the Johns Hopkins University, all rights reserved).

The CTCAE version 5.0 from The National Cancer Institute was used to evaluate paresthesia (grade 0–3), peripheral sensory neuropathy (grade 0–4) and peripheral motor neuropathy (grade 0–5) [25, 26]. Grade 0 equals no symptoms, and increasing score indicates increasing severity of symptoms with activities of daily living (ADL) being affected from grade 2.

The TNSc is a 7-item composite test, where each item is graded from 0 (normal) to 4 (severe neuropathy) given a total score from 0 to 28 [27–30]. Item 1–3 evaluates the patient’s sensory, motoric, and autonomic symptoms developed within the last week according to the original scoring manual. Item 4–7 test pin sensibility (using Neuropen®, Owen Mumford), vibration sensibility (by a Rydel-Seiffer c64 graduated tuning fork), muscle strength, and tendon reflexes. All tests are performed on both the upper and the lower limb.

An overview of the examinations is presented in Supplemental Fig. 1.

**Fig. 1:**
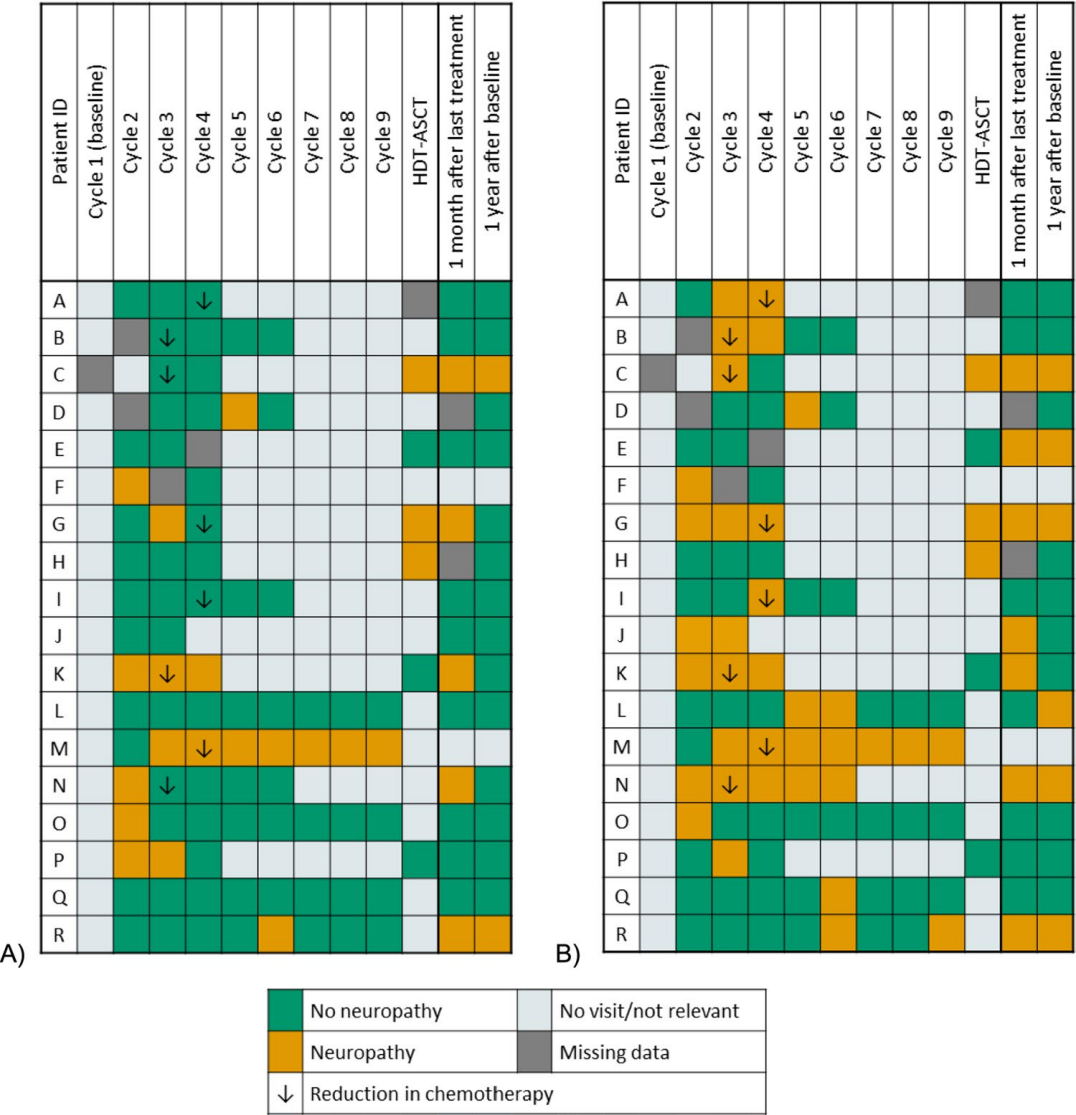
Neuropathy defined by the FACT/GOG-Ntx-13. Patients with a ≥ 10% drop in the neuropathy subscale of the FACT/GOG-Ntx-13 score either by A) the NTX-13 score or B) the NTX-4 score. “No visit/not relevant” reflect that the questionnaire has not been completed either due to the individual treatment plan or withdrawal from the study. The figure only includes patients that developed neuropathy quantified by the either the NTX-13 or NTX-4

### Statistical analysis

Data were collected and managed using REDCap [31, 32]. Baseline information was described by proportions (for categorical data) and medians and interquartile range (IQR) (for continuous data). Changes in FACT/GOG-Ntx-13 were presented by proportions (for reductions of ≥ 10%) and boxplots for selected visits including last cycle. Last cycle was defined as the last treatment with a neurotoxic therapy according to each patient’s treatment regimen and individual schedule. Differences between visits were tested using paired *t*-tests. All *p*-values were adjusted using the Holm-Bonferroni to handle multiple testing [33]. For calculation of paired *t*-tests missing data were imputed using multiple imputation by chained equations and predictive mean matching generating 1000 datasets with associated *p*-values, that was pooled using Rubin’s rules [34–36]. Imputations were performed using all data from the FACT/GOG-Ntx-13 and the treatment regimen. Any tingling and numbness in hands and feet were described using the questions Ntx1 and Ntx2 from the FACT/GOG-Ntx-13.

Results from the CTCAE were presented by proportions. The likelihood of being graded with a CTCAE score of 0 or ≥ 1 at baseline, cycle 3, last cycle, 1 month after treatment, or 1 year after baseline was tested by logistic regression with fixed and random effects to handle interpersonal variation. Missing values were imputed as stated above, and the odds ratios were pooled using Rubin’s rules. Associated *p*-value was calculated using Wald’s test and adjusted with the Holm-Bonferroni.

All statistical analyses were performed in R version 4.3.1 (R foundation for Statistical Computing, Vienna, Austria). All analyses were performed after end of study, and the scores were blinded to patients and the clinical staff during the study.

### Ethical approval

The study was approved by The North Denmark Region Committee on Health Research Ethics (N-20190068) and registered at Clinicaltrials.gov (Trial Registration Number: NCT04393363) on March 19, 2020, and in the research registry of the North Denmark Region (2020–081). This study was conducted in accordance with the principles of the Declaration of Helsinki and informed consent was obtained from all patients included in the study.

## Results

### Longitudinal measurements with a high degree of completeness

Sixty-six patients fulfilled the in- and exclusion criteria and were approached for participation. Twenty-five patients accepted and signed an informed consent. Two patients withdrew after the first visit due to their physical condition, leaving 23 patients with a total of 171 visits for analysis (Supplemental Fig. 2). Median number of visits for each patient was 7 (range 3–10). Six planned visits were not performed, resulting in 3.4% missing visits overall. However, it was not possible to conduct all examinations at each visit due to the patients’ condition, and the percentage of missing data ranged by examination from 5.1% (CTCAE) to 8.5% (TNSc).

**Fig. 2:**
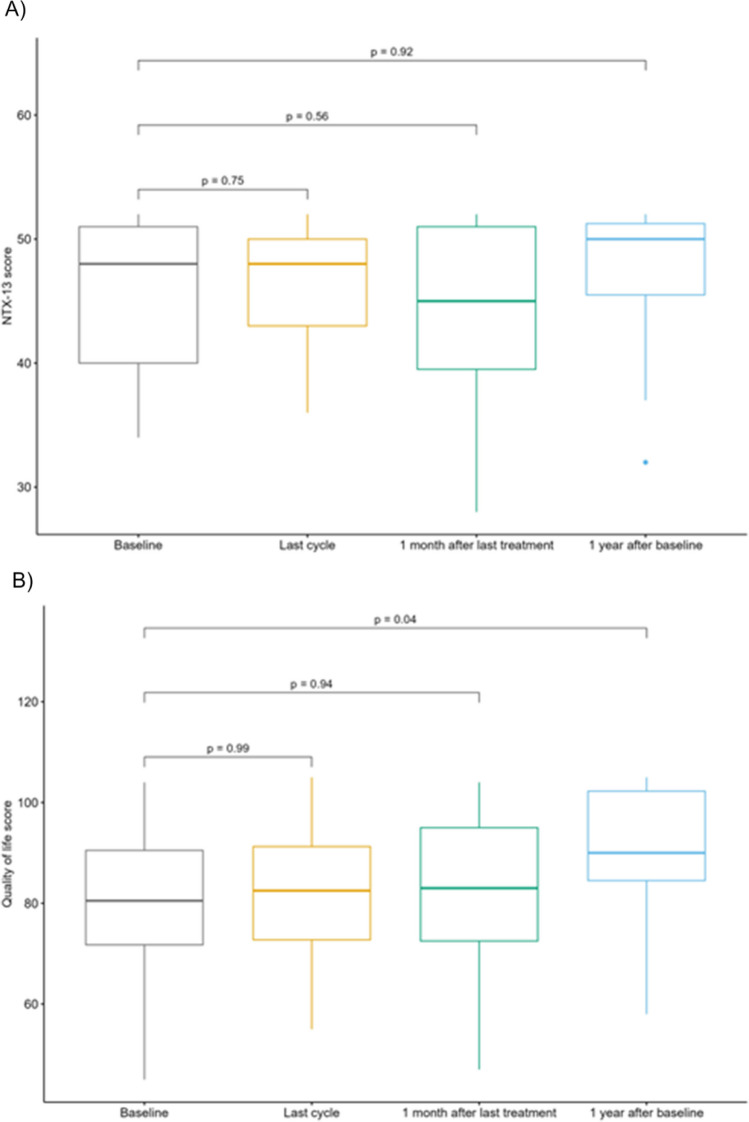
Changes in FACT/GOG-Ntx-13. Boxplot for subscores of A) neuropathy (NTX-13 score) and B) quality of life at different time points in treatment group by all patients or the different treatment regimens. Provided p-values are adjusted using Holm-Bonferroni. One patient was an outlier in the neuropathy score due to preexisting neuropathy and was exclude from this analysis

Nineteen (82.6%) patients with multiple myeloma were treated with either VRd (five patients), VRd and HDT-ASCT (nine patients), or D-VMP (five patients). The remaining four patients (17.4%) were diagnosed with lymphoma and treated with R-CHOP (Table 2). The median cumulative dose of neurotoxic chemotherapy for each regimen is listed in Supplemental Table 1. One patient without CIPN when entering the study was previously treated with bortezomib due to the present cancer. Three patients withdrew during the study (Supplemental Fig. 2).

**Table 2:** Baseline and clinical characteristics of patients

Age, median (IQR)	72 (64–76)
Sex, *n* (%)	
Male	9 (39.1)
Female	14 (60.9)
Diagnosis, *n* (%)	
Multiple Myeloma	19 (82.6)
Lymphoma	4 (17.4)
Treatment, *n* (%)	
R-CHOP	4 (17.4)
VRd	5 (21.7)
VRd and HDT-ASCT	9 (39.1)
D-VMP	5 (21.7)
Comorbidity, *n* (%)	
Diabetes Mellitus type 2	2 (8.7)
Smoking	
Never smoker	12 (52.2)
Former smoker	10 (43.5)
Current smoker	1 (4.3)
Weekly alcohol consumption, *n* (%)	
≤ 7 units	21 (91.3)
> 7 units	2 (8.7)

Abbreviations: *R-CHOP* rituximab, cyclophosphamide, doxorubicin, vincristine, and prednisolone; *VRd* bortezomib, lenalidomide, and dexamethasone; *HDT-ASCT* high-dose therapy and autologous stem-cell transplantation; *D-VMP* daratumumab, bortezomib, melphalan, and prednisolone

### Patient-assessed neuropathy detected in almost half of patients

Eleven patients (47.8%) experienced a reduction of ≥ 10% in the NTX-13 score reflecting presence of CIPN (Fig. 1A). Eight patients (34.8%) were reduced or changed in scheduled treatment due to symptoms of neuropathy during treatment. Only four of these eight patients (50%) had a ≥ 10% drop in total NTX-13 score related to their treatment alteration but all eight patients had a related drop when using the shortened NTX-4 score (Fig. 1B). In total, 18 patients (78.3%) had a reduction of ≥ 10% in the NTX-4 score. All treatment alterations were done right before either cycle 3 or 4. Four other patients were reduced in chemotherapy due to other reasons and none of them developed neuropathy.

The proportion of patients with any numbness and tingling in hands or feet changed throughout treatment. The highest proportion in hands was seen right before cycle 3 (36.4%) and at 1 month after last treatment (57.9%) in feet. For hands and feet combined the highest proportion was seen at cycle 6 (58.3%).

Comparing the changes in NTX-13 score there was no significant difference from baseline during follow-up to 1 year after baseline with median NTX-13 score changing from 48 (baseline) to 50 (1 year after baseline, Fig. 2A). Overall, the pattern was similar for changes in QoL score (Fig. 2B). However, the score increased significantly from baseline to 1 year after baseline (*p* = 0.04) with an increase in median score from 84 to 90 reflecting an increase in the patients’ QoL within this year.

### Paresthesia was the main symptom

The proportions of patients with a CTCAE score ≥ 1 for paresthesia increased significantly from baseline throughout follow-up until 1 month after last treatment (*p* = 0.019–0.045, Table 3). For both peripheral sensory neuropathy and peripheral motoric neuropathy there were no significant difference in scores during follow-up (*p* ≥ 0.173).

**Table 3:** Proportions of CTCAE score by time in treatment. Scores ≥ 1 equals symptoms, and scores ≥ 2 reflect symptoms that interferes with Activities of Daily Living (ADL). Provided p-values are adjusted using the Holm-Bonferroni

	Baseline	Cycle 3	Compared to baseline	Last cycle*	Compared to baseline	1 month after last treatment	Compared to baseline	1 year after baseline	Compared to baseline
	*N* (%)	*N* (%)	*p*-value	*N* (%)	*p*-value	*N* (%)	*p*-value	*N* (%)	*p*-value
Paresthesia (0–4)									
0	18 (78%)	10 (45%)		6 (30%)		9 (45%)		12 (60%)	
≥ 1	5 (22%)	12 (55%)	*0.045*	14 (70%)	*0.019*	11 (55%)	*0.039*	8 (40%)	0.086
1	5 (22%)	10 (45%)		13 (65%)		9 (45%)		8 (40%)	
2	0	1 (5%)		1 (5%)		1 (5%)		0	
3	0	1 (5%)		0		1 (5%)		0	
4	0	0		0		0		0	
NA	0	1		3		3		3	
Peripheral sensory neuropathy (0–3)									
0	21 (91%)	16 (73%)		17 (85%)		14 (70%)		17 (85%)	
≥ 1	2 (9%)	6 (27%)	0.191	3 (15%)	0.793	6 (30%)	0.181	3 (15%)	0.410
1	2 (9%)	5 (23%)		3 (15%)		4 (20%)		2 (10%)	
2	0	1 (5%)		0		2 (10%)		1 (5%)	
3	0	0		0		0		0	
NA	0	1		3		3		3	
Peripheral motoric neuropathy (0–5)									
0	17 (74%)	18 (82%)		16 (80%)		16 (80%)		18 (90%)	
≥ 1	6 (26%)	4 (18%)	> 0.99	4 (20%)	> 0.99	4 (20%)	0.774	2 (10%)	0.173
1	4 (17%)	2 (9%)		2 (10%)		3 (15%)		0	
2	2 (9%)	1 (5%)		2 (10%)		1 (5%)		2 (10%)	
3	0	1 (5%)		0		0		0	
4	0	0		0		0		0	
5	0	0		0		0		0	
NA	0	1		3		3		3	

Abbreviations: *CTCAE* Common Terminology Criteria for Adverse Events

^*^Last treatment was defined as the last treatment with a neurotoxic treatment given to each patient according to the treatment regimen and individually treatment schedule

**Fig. 3:**
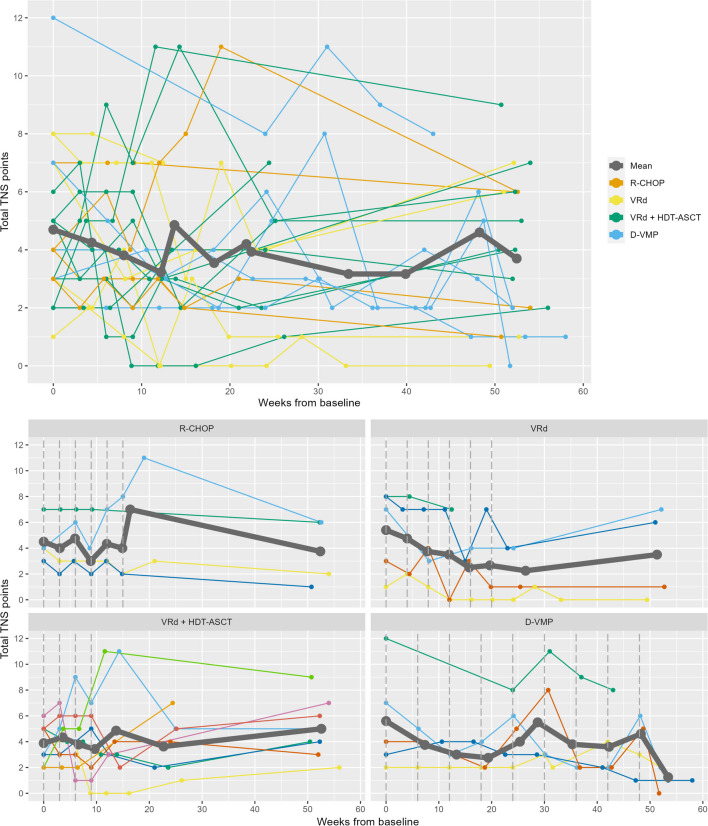
Changes in TNSc-score. Individual changes in TNSc score for all patients and grouped by treatment regimen with a mean for each group. The vertical dashed lines indicate when treatment is given based on the standard treatment plan. Abbreviations: TNSc: Total Neuropathy Score-clinical; R-CHOP: rituximab, cyclophosphamide, doxorubicin, vincristine, and prednisolone; VRd: bortezomib, lenalidomide, and dexamethasone; HDT-ASCT: high-dose therapy and autologous stem-cell transplantation; D-VMP: daratumumab, bortezomib, melphalan, and prednisolone

There were only small variations in the mean scores of the TNSc. Overall, no association was observed between treatment duration and the TNSc score (Fig. 3). Remarkably, the mean TNSc score for patients treated with VRd and D-VMP tended to decrease throughout treatment with two subsequently increases approximately mid-treatment and at the end of treatment for patients treated with D-VMP.

One patient had vitamin B12 deficiency 1 year after baseline (not present at baseline or 1 month after treatment). The deficiency was not related to neuropathy.

## Discussion

This longitudinal observational study describes the course of CIPN throughout treatment and after treatment for patients treated with vincristine, bortezomib, or lenalidomide. As recommended in the literature, CIPN was assessed at each contact using both patient-reported and clinician-assessed outcomes [8, 37, 38]. The Functional Assessment of Cancer Therapy/Gynecologic Oncology Group-Ntx-13 (FACT/GOG-Ntx-13), and the Total Neuropathy Score-clinical (TNSc) are suggested most suitable for measuring CIPN and were in the present study combined with the Common Terminology Criteria for Adverse Events (CTCAE), that is widely used in clinical settings [37, 39, 40].

Our primary outcome, a 10% decrease in the neuropathy subscale (NTX-13 score) of the FACT/GOG-Ntx-13 showed that overall, 47.8% of patients developed neuropathy at some point throughout treatment and follow-up. Using the shortened NTX-4 the prevalence increased to 78.3%. The majority developed neuropathy in the beginning of the treatment after only one or two cycles. Post-study comparison with the changes in treatment driven by neurological symptoms reported to the physicians showed a poor relation between reduction in NTX-13 and dose adjustments. A better relation was seen with the NTX-4, indicating that paresthesia was the dominant symptom. As the shortened version has a higher sensitivity it may be better to detect CIPN within this population. The NTX-4 combined with three other questions (Ntx-8, Ntx-9, and An6) were also found to be most informative in a study by Alberti et al. [41]. The drawback of using NTX-4 is the low specificity. However, the large group of “false positive” may reflect a group of patients that are in risk of developing neuropathy and who needs extra monitoring.

The proportion of any numbness and tingling in hands and feet estimated separately was highest before cycle 3 (36.4%) and 1 month after last treatment (57.9%), respectively. Two other studies have examined the proportion of numbness and tingling in hands and feet in longitudinal studies using the FACT/GOG-Ntx. Molassiotis et al. also found the highest proportion of numbness and tingling in feet after treatment in patients treated with taxanes- and platinum-based chemotherapy; however, only 46.3% had CIPN after end of treatment (6 months after enrollment) [14]. Contrary, to our findings Molassiotis et al. found the highest proportion (49.6%) of any numbness and tingling in hands at cycle 6 (corresponding to last cycle). Timmins et al. examined CIPN in patients treated with paclitaxel before, mid-treatment, and after treatment and found the highest proportion at end of treatment for both hands and feet (> 80%) [24]. The differences between the studies may be explained by the use of different chemotherapies.

The median score of the NTX-13 score did not significantly change throughout treatment. This could be due to neuropathy already presented before baseline. Many patients with multiple myeloma have neuropathy associated with paraproteinemia or the disease itself at diagnosis [15, 16, 42, 43]. This is supported by the drop in TNSc score after baseline observed among patients treated with D-VMP and VRd. This highlights the difficulty in assessing CIPN in patients treated with multiple myeloma, as they can experience neuropathy for a variety of reasons. We found an increase in the QoL score from baseline to 1 year after baseline (*p* = 0.04). This might not be surprising, as the patients at baseline may be stressed due to the newly diagnosis of cancer. The QoL score did not increase during treatment or at 1 month after last treatment, underlining the difficulty in going through cancer treatment. The results were similar to a study by Major et al., who found a significant difference in QoL from diagnosis to 1 year after diagnosis using the European Organization for Research and Treatment of Cancer (EORTC) QLQ-C30 [19].

The present study used patient-reported outcome to define the primary outcome. It has been widely discussed whether patient-reported outcome or clinician-assessed outcome is most suitable for measurement of CIPN. Patient-reported outcomes are easy to use both in clinical and research settings, but often yield a higher incidence of CIPN and are criticized for being inaccurate. Contrary, clinician-assessed outcomes are often criticized for not being able to capture the mildest cases of CIPN. We found the same pattern in the present study with the highest prevalence in patient-reported outcomes being 58.3% at cycle 6 and only 30.0% at 1 month after last treatment using the standard clinician-assessed CTCAE categories (peripheral sensory and motoric neuropathy). However, when including the CTCAE score for paresthesia the proportion was 70.0% at last cycle. Overall, the proportion of patients with a CTCAE-score ≥ 1 for paresthesia increased significantly from baseline until 1 month after last treatment; hereafter, it decreased to the same level as baseline. We did not find any differences in sensory peripheral neuropathy. This may reflect that even though patients have paresthesia the symptoms do not interfere with other sensory sensation. It may also indicate that the CTCAE score for paresthesia is better to detect the symptoms associated with CIPN than the standard CTCAE for peripheral sensory neuropathy. The proportion of CTCAE ≥ 1 for peripheral motoric neuropathy did not change significantly throughout treatment. It could reflect that motoric symptoms are not the predominantly problem in patients treated with vincristine, bortezomib, or lenalidomide, which is in agreement with the literature [1, 4]. Another explanation could be the high proportion of score ≥ 1 at baseline. This could reflect symptoms related to multiple myeloma as the proportion of patients with motoric neuropathy is much higher than the prevalence of peripheral neuropathy in the general population, that is only 2–8% depending on age [15, 16, 42–44]. It could also reflect a challenge with assessment of CIPN, as fatigue and weakness may be misclassified as peripheral motoric neuropathy when not examined by a neurologist [45, 46]. Finally, this study allowed patients to have some degree of neuropathy before inclusion to reflect a real-world setting, making it possible that some patients were affected by neuropathy for other reasons before inclusion.

The overall mean of the TNSc score decreases up until week 12 and increases until week 13 corresponding to cycle 3–5 or the time of the HDT-ASCT-treatment depending on the treatment regimen. The initial decrease is probably driven by a decrease in patients with multiple myeloma as discussed above. The peak in TNSc is seen later compared to the questionnaire FACT/GOG-Ntx-13, the clinician-assessed CTCAE, and the dose adjustments in chemotherapy regulated by the clinical staff. It may reflect that the TNSc only scores subjective symptoms within the first week of their appearance, so the later peak observed by the TNSc could reflect the onset of objective symptoms. Overall, the results from the TNSc showed small variations in mean scores making it difficult to detect neuropathy within our cohort. This could be due to the challenges with assessing CIPN in patients with multiple myeloma, a large interpersonal variation within our cohort, or the missing scoring of persistent symptoms using the TNSc.

The major strength of the study is the longitudinal design with repeated measurements for the patients throughout the whole treatment course and afterwards. This is further strengthened by the low grade of missing visits (3.4% overall). Another strength is the use of both patient-reported and clinician-assessed outcomes and the possibility to relate the findings with the treatment alterations.

The study is limited by the low number of included patients increasing the risk of finding no changes in neuropathy risk scores, when there actually is a difference (type II error). Due to the low number of included patients, no test of correlation between the included tests was performed. Finally, the inclusion of three different types of chemotherapy with potential different profiles of CIPN makes it difficult to draw conclusions for the individual chemotherapy.

In conclusion, this longitudinal observational study investigating the prevalence and course of CIPN in patients treated with vincristine, bortezomib, or lenalidomide found a neuropathy prevalence of 47.8% assessed by the questionnaire FACT/GOG-Ntx-13. A peak in CIPN was seen at two timepoints, approximately at cycle 3–4 of chemotherapy and at 1 month after last treatment. For a rapid screening of CIPN symptoms in a clinical setting we recommend using the four questions regarding sensory neuropathy (NTX-4) from the FACT/GOG-Ntx-13 questionnaire as this assessment has the closest relationship with the dose adjustments performed during treatment. Larger studies investigating the CIPN development in patients treated with vincristine, bortezomib, or lenalidomide separately are needed to fully understand the development of CIPN in these patients.

## Supplementary Information

Below is the link to the electronic supplementary material.

Supplementary file1 (DOCX 75 KB)

## Data Availability

Data is available on request from the corresponding author.
