# Supplementary material for: The course of chemotherapy-induced peripheral neuropathy (CIPN) in hematological patients treated with vincristine, bortezomib, or lenalidomide: the NOVIT study
Source: Support Care Cancer. 2025 Feb 26;33(3):225. doi: 10.1007/s00520-025-09282-3 (PMC11865131; doi:10.1007/s00520-025-09282-3)
Supplement: Supplementary file 1 — Supplementary file1 (DOCX 75 KB) [file 520_2025_9282_MOESM1_ESM.docx]

**Supplementals for**

***The course of chemotherapy-induced peripheral neuropathy (CIPN) in hematological patients treated with vincristine, bortezomib, or lenalidomide: The NOVIT study***

*Journal: Supportive Care in Cancer*

By Eva Futtrup Maksten*, Carsten Dahl Mørch, Lasse Hjort Jakobsen, Kristian Hay Kragholm, Pernille From Blindum, Mikkel Runason Simonsen, Niels Ejskjaer, Karen Dybkær, Henrik Gregersen, Jakob Madsen, Tarec C. El-Galaly, and Marianne Tang Severinsen

*Corresponding author, affiliated to Department of Hematology, Clinical Cancer Research Center, Aalborg University Hospital, Aalborg, Denmark and Department of Clinical Medicine, Aalborg University, Aalborg, Denmark. E-mail: efm@rn.dk

**Supplemental Table 1**

|  | Vincristine | Bortezomib | Lenalidomide | Treatment duration |
| --- | --- | --- | --- | --- |
| R-CHOP | 6 (5.75-7.5) mg/m^2^ | - | - | 18 weeks |
| VRd | - | 23.4 (15.9-23.4) mg/m^2^ | 1650 (1650-1650) mg/m^2^ | 24-32 weeks |
| VRd + HDT-ASCT | - | 19.6 (18.4-20.8) mg/m^2^ | 1263.5 (1225-1400) mg/m^2^ | 12 weeks |
| D-VMP | - | 46.8 (39.6-46.8) mg/m^2^ | - | 54 weeks |

Cumulative dose of neurotoxic chemotherapy grouped by chemotherapy regimen quoted as median (IQR). Abbreviations: R-CHOP: rituximab, cyclophosphamide, doxorubicin, vincristine, and prednisolone; VRd: bortezomib, lenalidomide, and dexamethasone; HDT-ASCT: high-dose therapy and autologous stem-cell transplantation; D-VMP: daratumumab, bortezomib, melphalan, and prednisolone.

**Supplemental Figure 1**

**
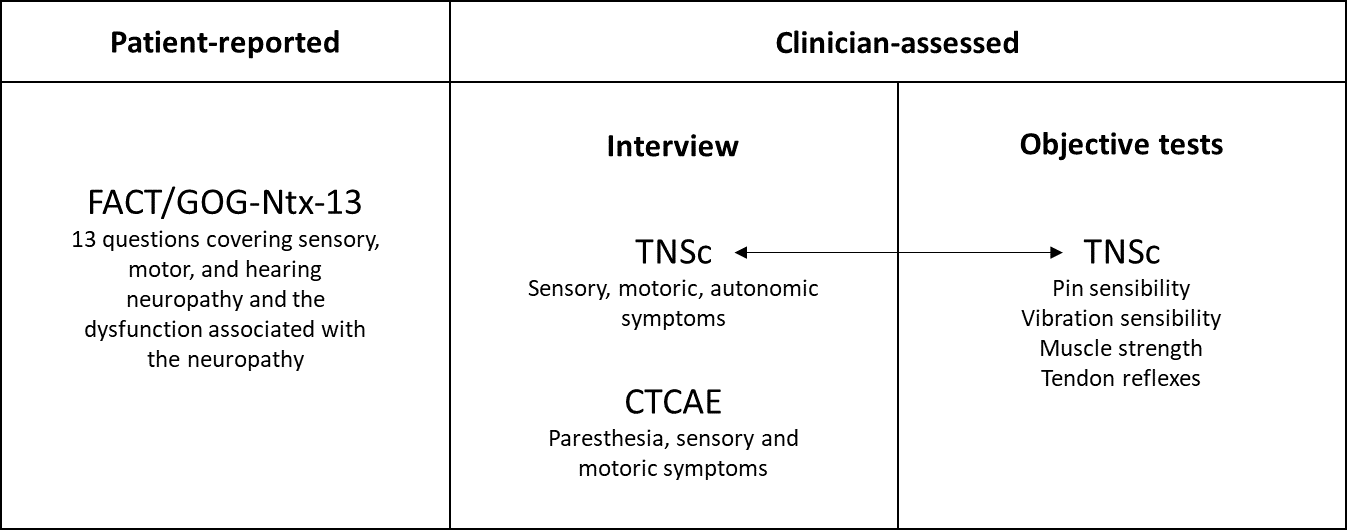
**

Overview of included examinations.

**Supplemental Figure 2**


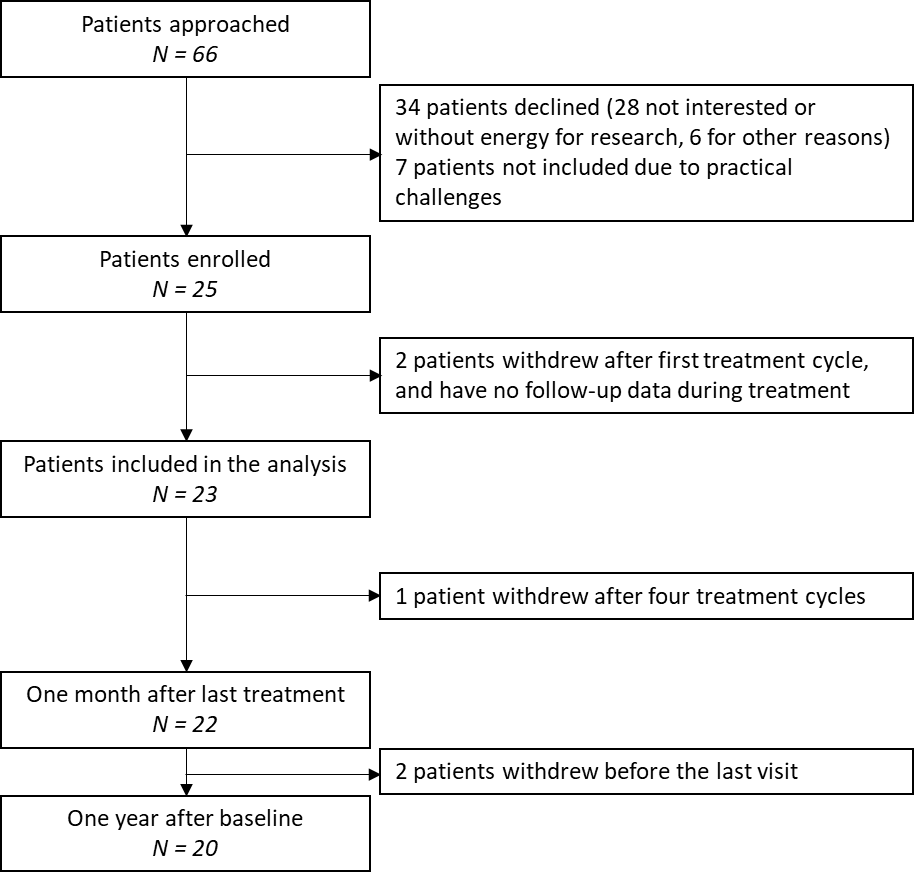


Consort diagram for the NOVIT study.
